# Prevalence of anxiety symptoms in infertile men: a systematic review and meta-analysis

**DOI:** 10.1186/s12889-024-19299-8

**Published:** 2024-07-06

**Authors:** Masoumeh Simbar, Vida Ghasemi, Reza Taherian, Mehri Kalhor, Fateme Mohammadian, Zahra Kiani

**Affiliations:** 1 Midwifery and Reproductive Health Research Center, Department of Midwifery and Reproductive Health, School of Nursing and Midwifery, Shahid Beheshti University of Medical Sciences, Tehran, Iran; 2 Department of Public Health, Asadabad School of Medical Sciences, Asadabad, Iran; 3 Department of Biostatistics, School of Allied Medical Sciences, Shahid Beheshti University of Medical Sciences, Tehran, Iran; 4 Department of Midwifery and Reproductive Health, Midwifery and Reproductive Health Research Center, School of Nursing and Midwifery, Shahid Beheshti University of Medical Sciences, Tehran, Iran; 5 Department of Midwifery and Reproductive Health, School of Nursing and Midwifery, Shahid Beheshti University of Medical Sciences, Tehran, Iran; 6 Midwifery and Reproductive Health Research Center, Shahid Beheshti University of Medical Sciences, Tehran, Iran

**Keywords:** Men, Infertility, Anxiety, Prevalence, Systematic, Meta-analysis

## Abstract

**Background:**

Infertility in men causes problems in various aspects of their lives, including personal, family and social life. One of the most important of these problems is anxiety. Anxiety in infertile men can affect their health, quality of life, and response to treatment, highlighting the significance of anxiety in these men. Thus, this systematic review and meta-analysis was conducted to investigate the prevalence of anxiety symptoms in infertile men.

**Methods:**

To conduct this review study, two researchers independently searched international databases such as PubMed, Cochrane Library, Web of sciences, Scopus, PsyINFO, and the Google scholar search engine in English without considering any time limit until January 2, 2024. Keywords such as "anxiety," "infertility," "prevalence," and "epidemiology" were used, taking into account the specific search method of each database. Using the Newcastle–Ottawa Scale (NOS), the quality of the articles was evaluated by two researchers independently.

**Results:**

In the systematic part of the study, 27 studies were included, and given the variety of measurement tools (8 different tools) used to investigate anxiety symptoms in infertile men, 24 studies were analyzed in five subgroups of tools. The pooled prevalence of anxiety symptoms in infertile men was 21.37% (95% CI: 15.73–27.02). The lowest and highest prevalence of anxiety in infertile men were related to the Beck anxiety inventory (BAI) and Depression Anxiety Stress Scales (DASS), accounting for 7.08% (95% CI: 3.27–10.90) and 34.90% (95%CI: 28.90–40.90) values respectively. This prevalence was 19.80% (95%CI: 9.01–30.59) for the Hospital Anxiety and Depression Scale (HADS), 30.06% (95%CI: 18.59–41.52) for the Spielberger Trait Anxiety Inventory (STAI-T), and 18.52% (95%CI: 7.76–29.29) for the Self-Rating Anxiety Scale (SAS).

**Conclusion:**

The results of this systematic review and meta-analysis indicated that the prevalence of anxiety symptoms in infertile men requires special attention to healthcare planning. The healthcare system of different countries should evaluate the symptoms of anxiety in infertile men and take appropriate measures to reduce them according to the culture of the countries. It is recommended that all infertile couples be assessed for anxiety symptoms using a standardized tool during their initial evaluation.

**Supplementary Information:**

The online version contains supplementary material available at 10.1186/s12889-024-19299-8.

## Introduction

Defined as the inability to conceive after at least one year of unprotected intercourse, infertility is a complex issue [1]. According to the latest global statistics in 2023, the prevalence of infertility in the world has been reported to be approximately 17.5%. However, this rate has been reported to be between 4 and 39.7% in different areas of the world [2], and the male factor is the main or effective factor in 50% of couples [3].

Infertility, as one of the main reproductive health problems, is a serious issue for the World Health Organization (WHO), because the lack of attention to it in different countries has led to psychological problems at the individual and social levels [4]. Infertility often causes a variety of social, psychological, physical and financial stresses [5]. In examining the negative psychological, behavioral and social consequences of infertility for both couples, it has been found that infertile couples experience a wide range of negative emotions, including anxiety, fear, avoidance, depression, guilt, and frustration [6].

Anxiety is a natural adaptive response of the body to stressful events such as infertility. Anxiety is the most common mental health problem associated with infertility, and studies show that anxiety remains high throughout the infertility diagnosis and treatment cycle [7]. Infertile individuals are almost twice as likely as other people to suffer from anxiety [8]. The incidence of mental disorders in male and female infertility is 12.41% after 2 years of diagnosis, and infertile couples may require psychological support during the diagnosis and treatment of infertility [9].

Some studies have indicated that men have a more negative response to infertility than women [10]. Infertile men, like many infertile women, suffer from anxiety, isolation, self-blame, and feelings of sexual inadequacy [11]. Because of the connection between male fertility and sexual power, infertile men often feel that infertility leads to an incomplete identity and masculinity [12].

Moreover, the inability to have children causes psychological problems for infertile men, especially in societies where fertility is highly valued and fertility is one of the basic goals of marriage. Infertility can lead to fear of rejection, divorce, remarriage, and many unpleasant changes for infertile men and cause them anxiety [13]. Therefore, psychological assessment of infertile individuals may contribute to more efficient use of health services and reduce the negative effects of anxiety on fertility, thereby increasing the success of infertility treatment [14].

Based on reports from different parts of the world, the prevalence of anxiety in infertile men varies significantly. However, the prevalence of anxiety in infertile men has not been reported in any meta-analysis. One of the objectives of meta-analysis is to provide accurate and valid information from a large sample size by integrating studies. This process provides accurate data that can help clinicians and service providers design interventions and treatment strategies [15]. Therefore, this study examines the prevalence of anxiety in infertile men through a systematic review and meta-analysis with the aim of highlighting the significance of psychological evaluation as an essential component of the treatment process for infertile men.

## Methods

### Search Strategy

To conduct this systematic review, two researchers independently searched international databases such as PubMed, Cochrane Library, Web of sciences, Scopus, PsyINFO, and the Google scholar search engine in English without considering any time limit until January 2, 2024. Keywords such as "anxiety," "infertility," "prevalence," and "epidemiology" were used along with "AND" and "OR" operators, taking into account the specific search method of each database (Search Strategy Appendix 1). Our PROSPERO registration number is CRD42024497844.

### Inclusion and exclusion criteria

Inclusion criteria were as follows: studies conducted on infertile men (defined as the absence of pregnancy after one year of sexual intercourse without the use of contraception), a minimum sample size of 30, reporting the prevalence of anxiety in infertile men using cross-sectional studies, using cross-sectional data from longitudinal studies, and investigating the prevalence of anxiety in infertile men using valid and standardized tools, such as reliable questionnaires or clinical interviews.

Exclusion criteria included the studies conducted on infertile men that did not report the prevalence of anxiety in infertile men, studies focusing on other mental and physical illnesses, history of psychiatric disorders means studies including participants with a history of psychiatric disorders, review articles, studies not written in English language, studies involving non-human samples, case reports, and studies for which the full text was not available.

### Outcome measures

The main outcome of this study was the prevalence of anxiety symptoms in infertile men, investigated by standard tools such as clinical interview or valid and reliable questionnaires.

### Data Extraction

The initial search for articles was conducted independently by two reviewers; the articles were entered into Endnote software, and duplicate articles were identified and removed. The titles, abstracts, and full texts of the articles were reviewed based on the inclusion criteria and then the articles were selected for data extraction. The data of the studies were extracted independently by two trained reviewers, and a third reviewer was consulted in case of disagreement.

**Fig. 1:**
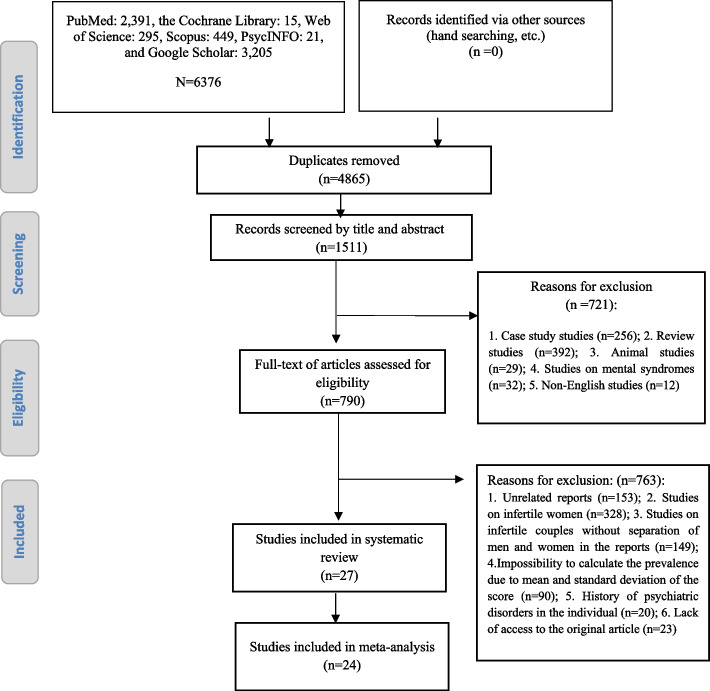
Flowchart for selection of studies

Required information, including author name, year of publication, place of research, sample size, type of infertility, prevalence of anxiety in infertile men, mean age, duration of infertility and type of instrument, was extracted from the studies. The review steps are described in Fig. 1.

### Quality Evaluation

The Newcastle–Ottawa Scale (NOS) checklist for assessing the quality of non-randomized trials in meta-analyses, modified by Zhang et al., was used for quality evaluation [16]. This checklist consists of 5 sections covering sample representativeness, sample size, non-respondents, anxiety assessment, and quality of descriptive statistics reporting. Accordingly, the quality of articles that met the inclusion criteria was assessed and scored from 0 to 5 using the NOS, and based on total scores of less than 3, 3 and more, respectively, were classified into two high-risk and low-risk groups. The quality assessment in this study was performed independently by two reviewers (ZK and FM), and a third reviewer was consulted in case of disagreement between the researchers. The results of the quality assessment of the studies are available in Appendix 2. The coefficient of agreement between the researchers was K = 0.88. This systematic review was reported based on the preferred reporting items for systematic reviews and meta-analyses (PRISMA) [17].

### Statistical analysis

In this review, the I^2^ index was used to assess heterogeneity among studies, and Egger's test was used to assess publication bias. In order to increase the validity of the study, at least 3 studies from each subgroup were used to the pooled prevalence. The subgroup analysis was performed based on tool type. Analysis was performed using IBM SPSS Statistics, version 29.0.1.0 (171). The significance level for statistical tests was set at 0.05.

### Ethics approval and consent to participate

Ethical approval was obtained from the Ethics Committee, Faculty of Pharmacy, Nursing and Midwifery, Shahid Beheshti University (Ethical code: IR.SBMU.RETECH.REC.1402.497). All methods were performed in accordance with relevant guidelines and regulations.

## Results

Initially, 6,376 articles were identified. After removing duplicate articles, the titles and abstracts of the articles were reviewed, and after discarding the articles unrelated to the purpose of the research and based on the inclusion criteria, the original text of 790 articles was reviewed, and then 27 articles were included in the systematic review and 24 articles in meta-analysis (Fig. 1).

The total sample size of the studies was 6,624 infertile men, with the smallest and largest sample size being 40 and 1,247 subjects, respectively. The lowest prevalence was 3.7% in Canada and the highest was 42.62% in Iran (Table 1). These studies used a variety of standard measurement tools to assess anxiety symptoms in infertile men. One study had used the Mini International Neuropsychiatric Interview (MINI), one study used the Primary Care Evaluation of Mental Disorders (PRIME-MD), one study Symptom Assessment-45 Questionnaire (SA-45), three studies used the Depression Anxiety and Stress Scale (DASS), three studies the Self-Rating Anxiety Scale (SAS), seven studies used the Hospital Anxiety and Depression Scale (HADS), six studies used the Spielberger Trait Anxiety Inventory (STAI-T), and five studies used the Beck Anxiety Inventory (BAI)

**Table 1:** Characteristics of the studies selected for the systematic

ID	Authors	countries	Sample size	Type of infertility	Prevalence of anxiety	Age (Y) (mean ± SD)	Mean years of infertility (mean ± SD)	Type of Tools	Quality Evaluation*
1	(Yang et al., 2017) [18]	China	771	Primary-secondary	7.80	32.30 ± 5.60	NA	STAI-T	4
2	(Vellani et al., 2013) [19]	Italy	94	Primary-secondary	40.40	NA	NA	STAI-T	3
3	(Schaller et al., 2016) [20]	Germany	82	Primary-secondary	41.44	37.22 ± 5.30	NA	STAI-T	3
4	(Zurlo et al., 2020) [21]	Italy	254	Primary-secondary	34.60	35.60 ± 3.79	3.27 ± 2.6.4	STAI-T	4
5	(Band et al., 1998) [22]	UK	130	Primary-secondary	39.00	36.52 ± 5.8 0	4.30 ± 2.20	STAI-T	3
6	(Shafierizi et al., 2022) [23]	Iran	40	Primary-secondary	18.90	32.57 ± 5.06	NA	STAI-T	4
7	(Zhang et al., 2022) [24]	China	1247	Primary-secondary	8.70	33.33 ± 5.88	3.50 ± 2.59	SAS	5
8	(Chen et al., 2016) [25]	China	202	Primary-secondary	27.20	31.69 ± 4.35	NA	SAS	3
9	(Liu et al., 2021) [26]	China	247	Primary-secondary	20.65	31.72 ± 4.86	3.10 ± 1.21	SAS	4
10	(Kooli et al., 2023) [11]	Tunisia	282	Primary-secondary	21.60	37.00 ± 6.00	NA	HADS	4
11	(Fernandes et al., 2023) [27]	Portugal	63	Primary-secondary	7.90	35.50 ± 5.50	5.00 ± 1.21	HADS	5
12	(Madero et al., 2017) [28]	Spain	201	Primary-secondary	8.50	41.60 ± 5.90	NA	HADS	4
13	(El Kissi et al., 2013) [10]	Tunisia	100	Primary-secondary	12.00	38.74 ± 5.87	5.19 ± 4.62	HADS	3
14	(Maroufizadeh et al., 2015) [29]	Iran	122	Primary-secondary	42.62	33.90 ± 5.87	6.20 ± 4.10	HADS	4
15	(Maroufizadeh et al., 2018) [30]	Iran	479	Primary-secondary	38.00	31.37 ± 5.69	5.62 ± 4.03	HADS	3
16	(Anderson et al., 2003) [31]	UK	113	Primary-secondary	8.90	NA	NA	HADS	3
17	(Anh et al., 2023) [32]	UK	385	Primary-secondary	33.30	33.70 ± 5.30	3.10 ± 1.21	DASS	3
18	(Musa et al., 2014) [33]	Malaysia	123	Primary-secondary	30.10	NA	NA	DASS	4
19	(Samani et al., 2017) [34]	Iran	180	Primary-secondary	41.33	32.94 ± 4.74	3.12 ± 2.59	DASS	3
20	(Chachamovich et al., 2010) [35]	Canada	162	Primary-secondary	3.70	36.10 ± 7.69	9.13 ± 4.72	BAI	3
21	(Peterson et al., 2007) [36]	Canada	295	Primary-secondary	7.00	34.50 ± 5.70	NA	BAI	4
22	(Öztekin et al., 2020) [9]	Turkey	130	Primary-secondary	17.00	29.95 ± 4.37	3.02 ± 2.34	BAI	3
23	(Drosdzol and Skrzypulec, 2009) [37]	Poland	188	Primary-secondary	4.79	31.40 ± 4.70	NA	BAI	5
24	(Klemetti et al., 2010) [38]	Finland	99	Primary-secondary	6.06	37.80 ± 2.50	NA	BAI	5
25	(Alosaimi et al., 2015) [39]	Saudi Arabia	176	Primary-secondary	20.50	NA	5.40 ± 4.90	MINI	4
26	(Volgsten et al., 2010) [40]	Sweden	412	Primary-secondary	4.90	NA	NA	PRIME-MD	4
27	(Haimovici et al., 2018) [41]	USA	47	Primary-secondary	17.00	36.00 ± 4.49	4.21 ± 3.25	SA-45	3

^*^Low risk of bias (≥ 3 points) and high risk of bias (< 3 points). Abbreviations: *NA* Not reported, *STAI-T* Spielberger Trait Anxiety Inventory, *MINI* Mini International Neuropsychiatric Interview, *HADS* Hospital Anxiety and Depression Scale, *BAI* Beck anxiety inventory, *DASS* Depression Anxiety Stress Scales, *SAS* The Self-Rating Anxiety Scale, *PRIME-MD* Primary Care Evaluation of Mental Disorders, *SA-45* Symptom Assessment-45 Questionnaire

### Evaluation of heterogeneity and *meta*-analysis

**Fig. 2:**
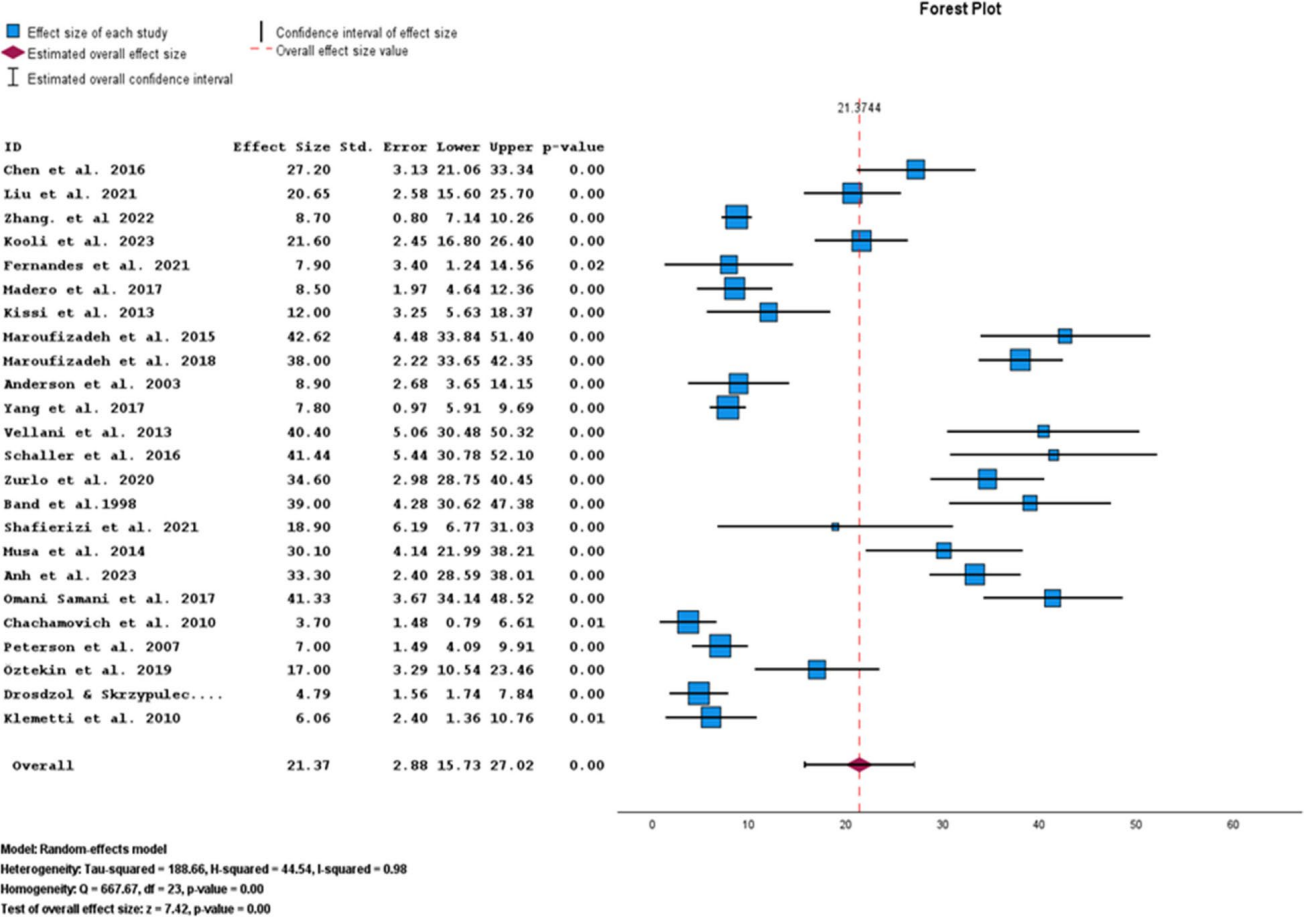
Pooled prevalence of anxiety symptoms in infertile men

The pooled prevalence of anxiety symptoms in infertile men was 21.37% (95% CI: 15.73–27.02) (Fig. 2).

**Fig. 3:**
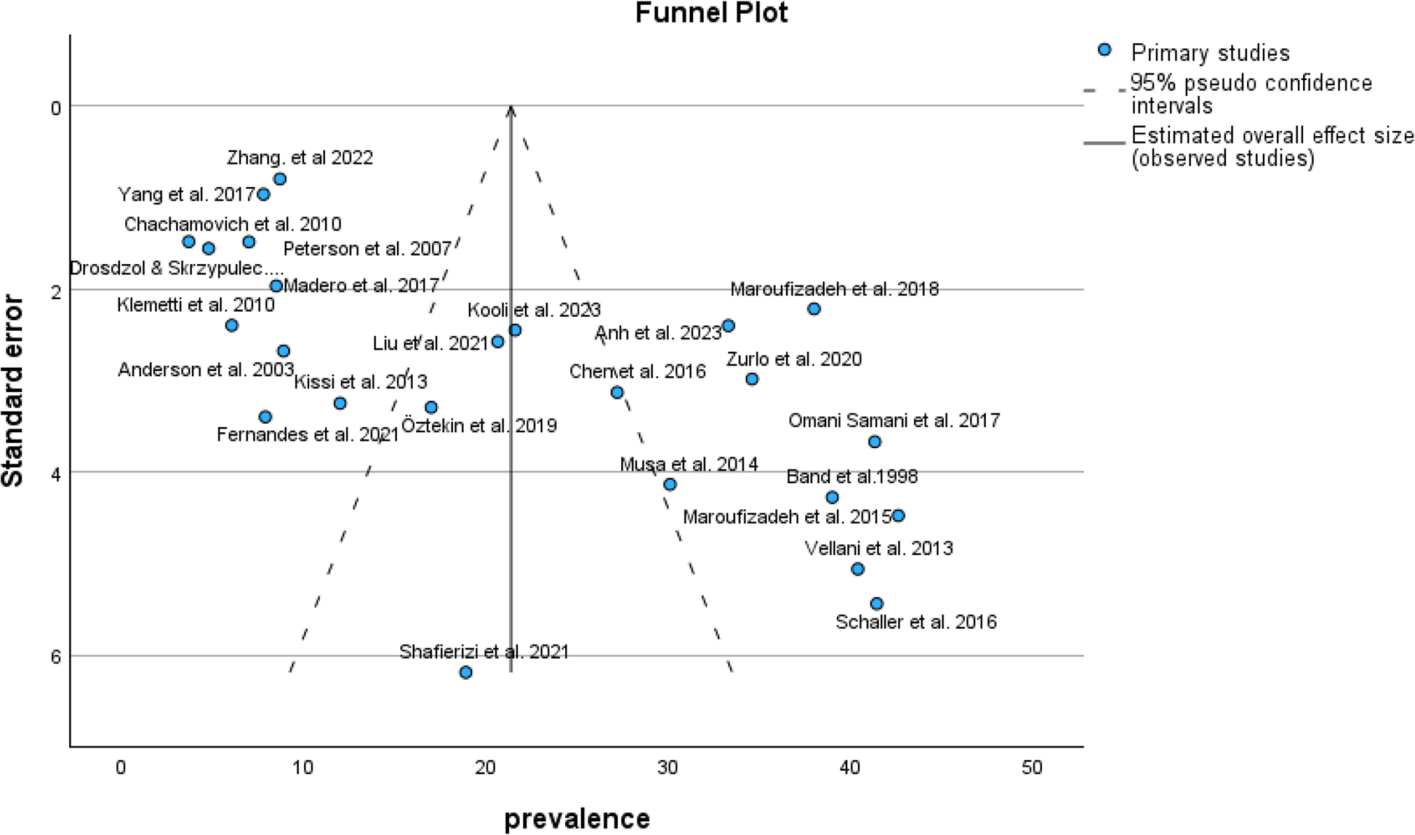
Funnel plot of pooled prevalence of anxiety symptoms in infertile men

The results of the funnel plot are also shown in Fig. 3. The results of Egger's regression test, with a t-value of t = 0.373 and *p*-value = 0.713, indicate no significant evidence of publication bias.

Given the fact that different tools have been used to investigate anxiety symptoms in infertile men, from the 27 articles in the systematic review section, 24 studies were used for the meta-analysis in 5 subgroups of tools (HADS, STAI-T, BAI, SAS, and DASS). The lowest and highest prevalence of anxiety in infertile men were related to the BAI and DASS tools, accounting for 7.08% (95% CI: 3.27–10.90) and 34.90% (95%CI: 28.90–40.90), respectively. This prevalence was 19.80% (95%CI: 9.01–30.59) for the HADS, 30.06% (95%CI: 18.59–41.52) for the STAI-T, and 18.52% (95%CI: 7.76–29.29) for the SAS (Table 2).

**Table 2:** Total and subgroup prevalence for anxiety symptoms in infertile men based on tools

Tools	Prevalence
BAI	7.08% (95% CI: 3.27–10.90)
SAS	18.52% (95%CI:7.76–29.29),
HADS	19.80% (95%CI: 9.01–30.59),
STAI-T	30.06% (95%CI:18.59–41.52
DASS	34.90% (95%CI: 28.90–40.90)
Total	21.37% (95% CI: 15.73–27.02)

### HADS

The 14-item HADS questionnaire was developed by Zigmond & Snaith (1983) to screen for symptoms of depression and anxiety in outpatient clinics of public hospitals. This questionnaire consists of 7 questions related to depression and 7 questions related to anxiety, and participants answer the questions using a 4-point Likert scale (0 to 3). The maximum possible score for each dimension is 21 [42]. Generally, scores of 0 to 7 on this questionnaire are considered to be normal, scores of 8 to 10 indicate mild symptoms, 11 to 14 indicate moderate symptoms, and 15 to 21 indicate severe symptoms [43]. The questionnaire has been translated into several languages, has psychometrically been evaluated and its validity and reliability have been confirmed [44–47].

**Fig. 4:**
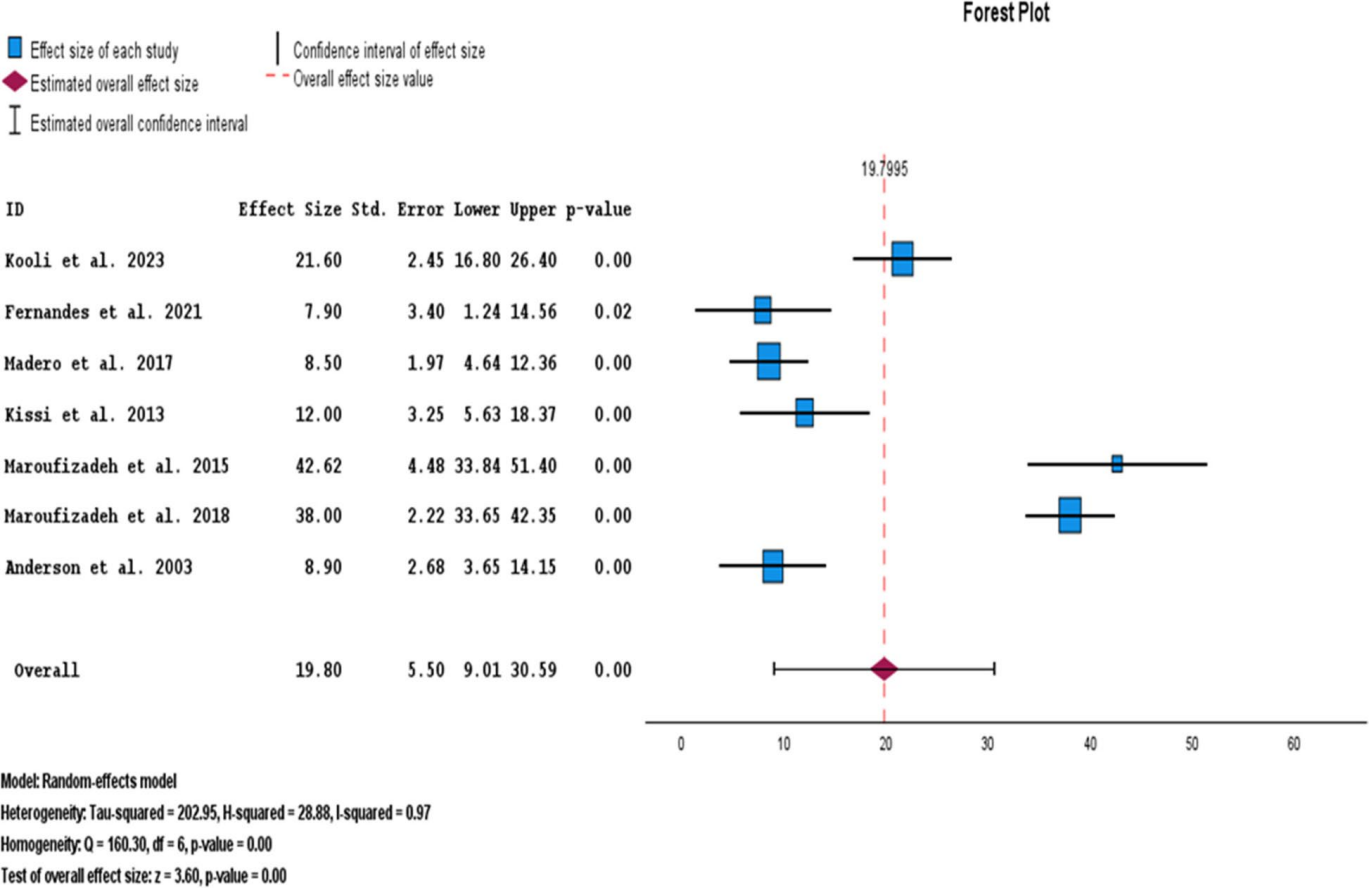
Pooled prevalence of anxiety symptoms in infertile men of the HADS subgroup

The subgroup analysis of the HADS tool indicated that the pooled prevalence of anxiety symptoms in men was 19.80% (95% CI: 9.01–30.59) (Fig. 4).

**Fig. 5:**
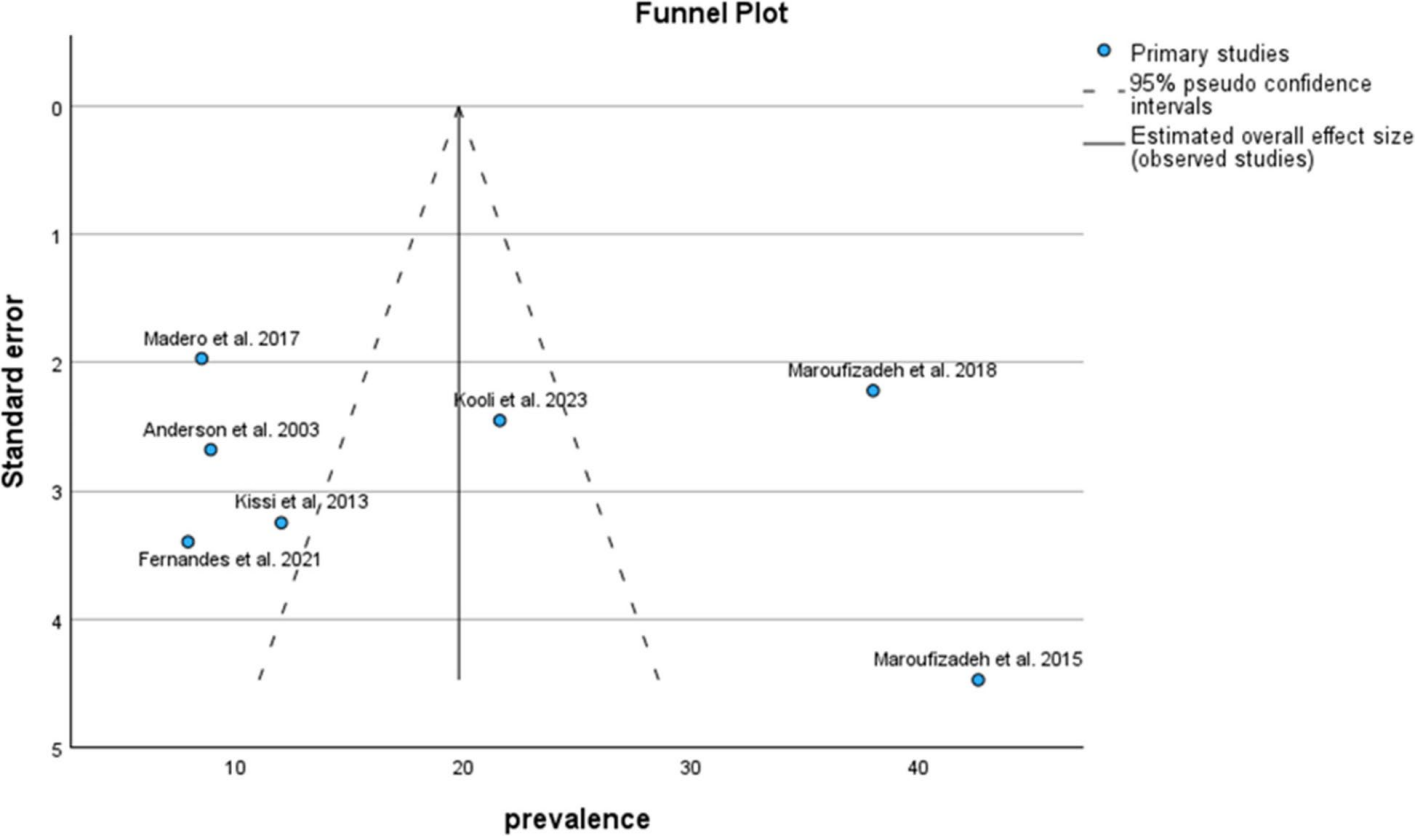
Funnel plot of pooled prevalence of anxiety symptoms in infertile men of the HADS subgroup

The results of the funnel plot are also shown in Fig. 5. The results of Egger's regression test, with a t-value of 0.130 and *p*-value = 0.902, suggest no significant evidence of publication bias.

### STAI-T

The Spielberger State-Trait Anxiety Inventory (STAI-T) is a 40-item self-report questionnaire. It is designed to assess both state anxiety (the respondent's current temporary state) and trait anxiety (the general tendency to experience anxiety). Each section of the inventory contains 20 questions, for a total of 40 items [48]. The inventory was originally developed by Spielberger, Gorsuch, and Lushene in 1970 as a tool for assessing anxiety [49]. It consists of 40 items, with response options ranging from "not at all / almost never" (1), somewhat/ sometimes (2), moderately so/ often (3), and very much so/ almost always (4). It has been widely used in numerous studies in different regions of the world and has shown good validity and reliability [50, 51].

**Fig. 6:**
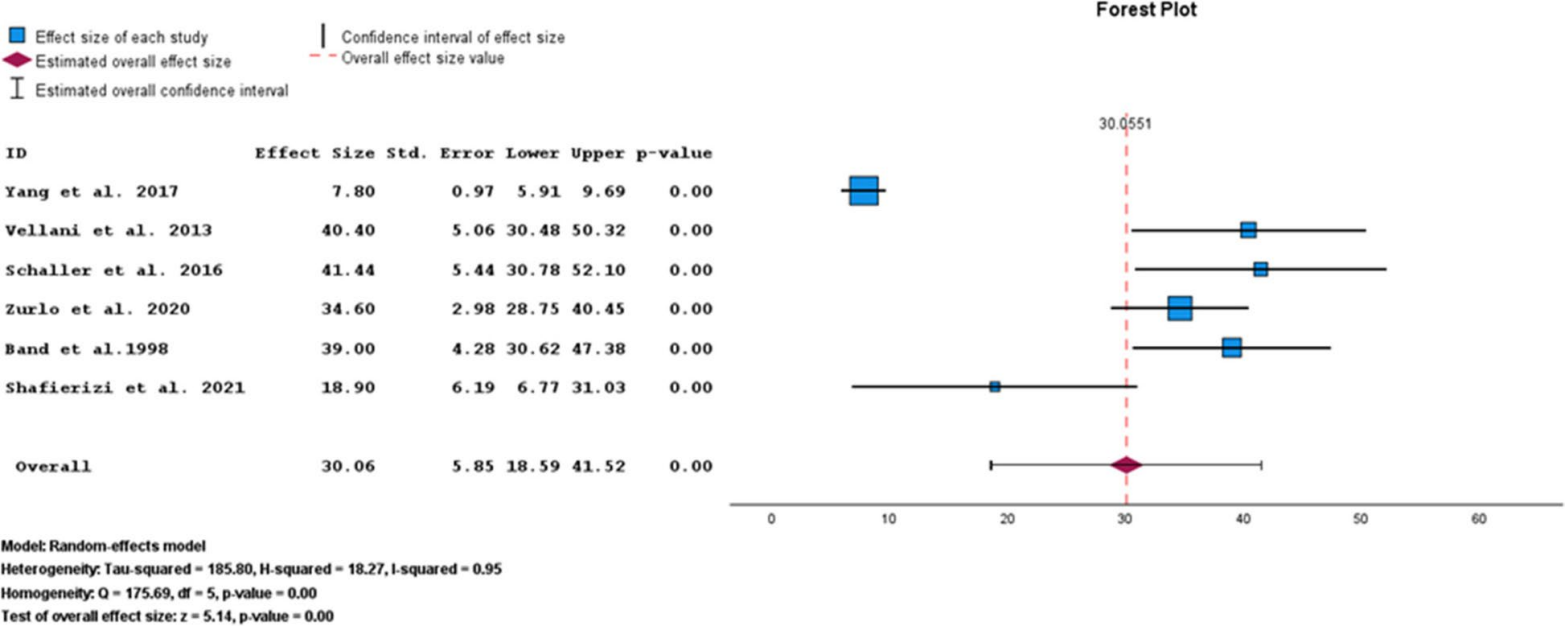
Pooled prevalence of anxiety symptoms in infertile men of the STAI-T subgroup

Based on the STAI-T subgroup results, the pooled prevalence of anxiety symptoms in infertile men was 30.06% (95% CI: 18.59–41.52) (Fig. 6).

**Fig. 7:**
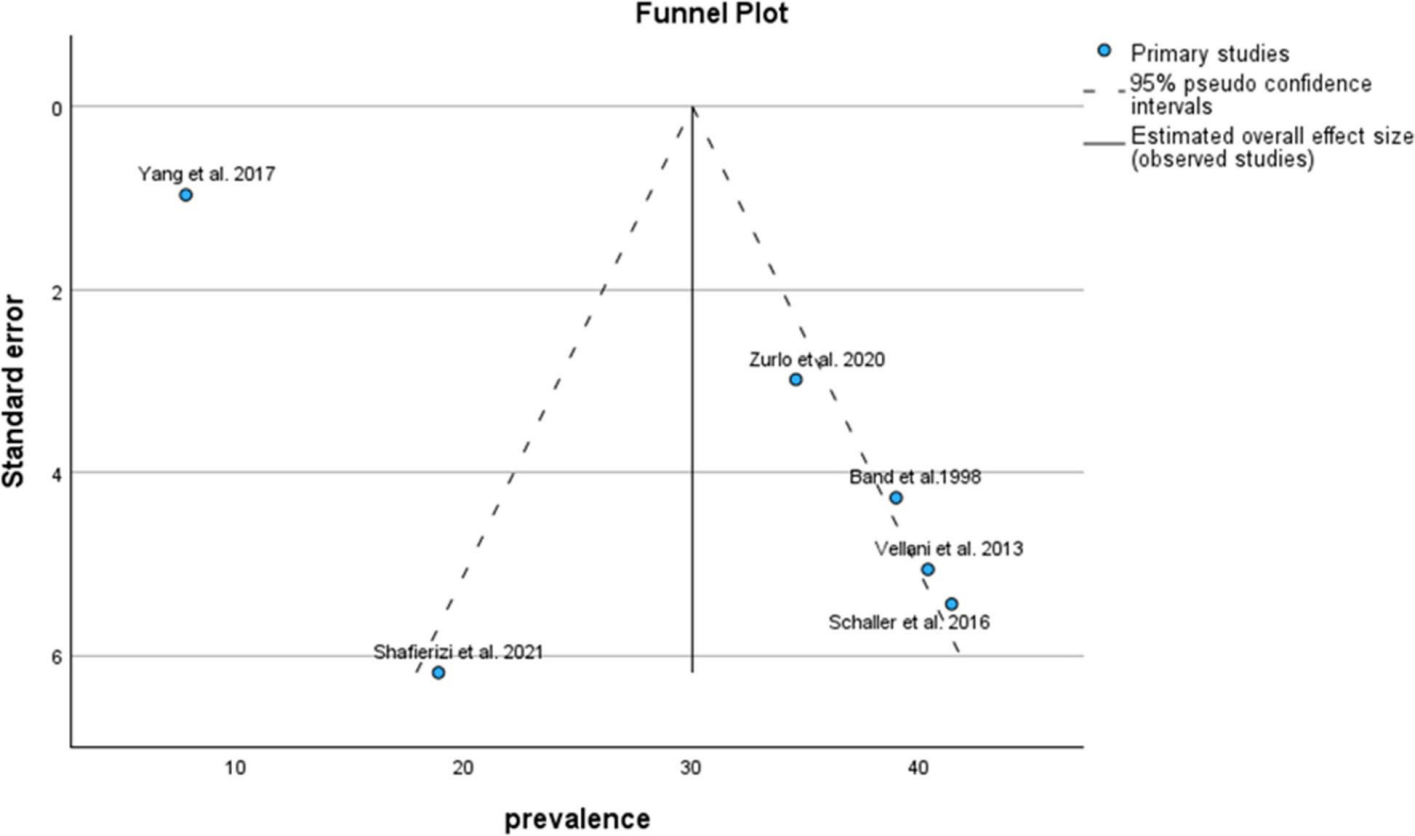
Funnel plot of the pooled prevalence of anxiety symptoms in infertile men of the STAI-T subgroup

The results of the funnel plot are also shown in Fig. 7. The results of Egger's regression test, with a t-value of 1.030 and *p*-value = 0.361, indicate no significant evidence of publication bias.

### BAI

This questionnaire, developed by Beck et al. in 1988, consists of 21 questions related to anxiety symptoms. All questions in the questionnaire are rated on a 4-point Likert scale, with the following options: "Not at all" (0), " Slightly, but it didn't bother me much" (1), "Moderately—it was not pleasant at times" (2), and "Severely – it bothered me a lot" (3). The total score is calculated by summing up the scores of all 21 items. Scores between 0 and 21 on this questionnaire indicate low levels of anxiety, scores between 22 and 35 indicate moderate levels of anxiety, and scores of 36 and above indicate potentially higher levels of anxiety. Cronbach's alpha was reported to be 0.91 in the original version of the questionnaire and 0.75 in the open test [52]. The validity and reliability of this questionnaire have been confirmed in several studies [53–55].

**Fig. 8:**
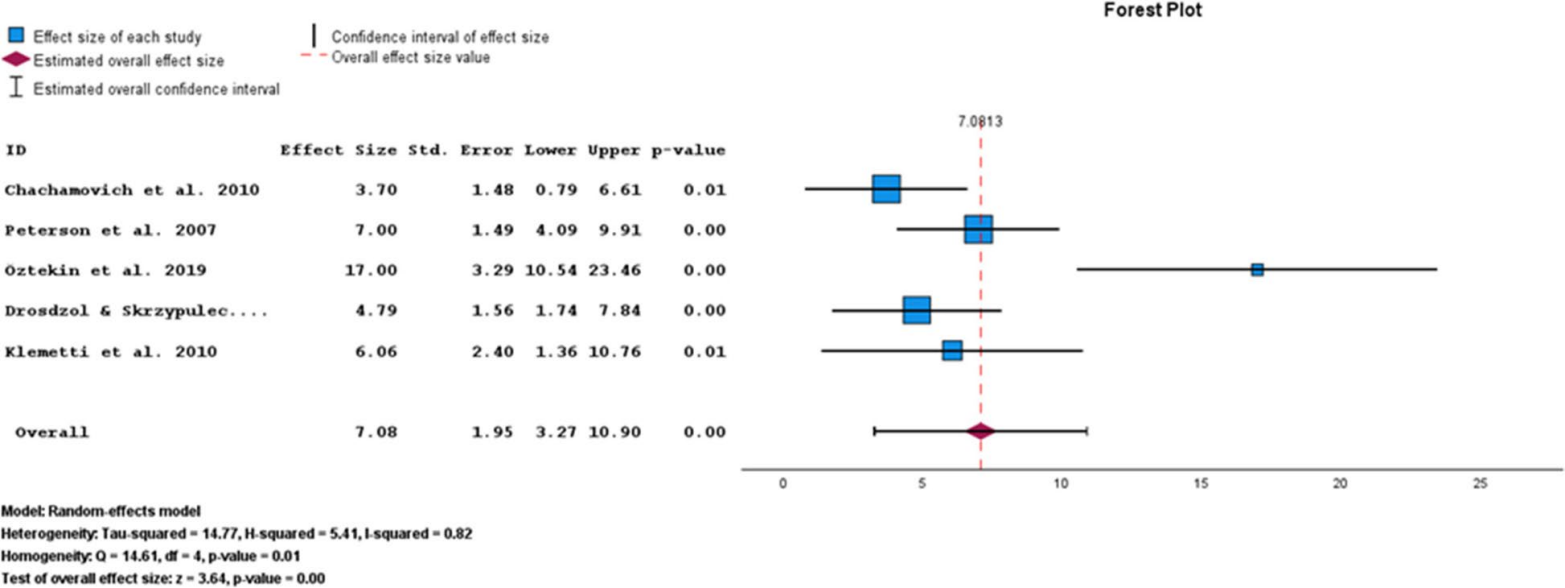
Pooled prevalence of anxiety symptoms in infertile men of the BAI subgroup

Based on the BAI subgroup results, the pooled prevalence of anxiety symptoms in infertile men was 7.08% (95% CI: 3.27–10.90) (Fig. 8).

**Fig. 9:**
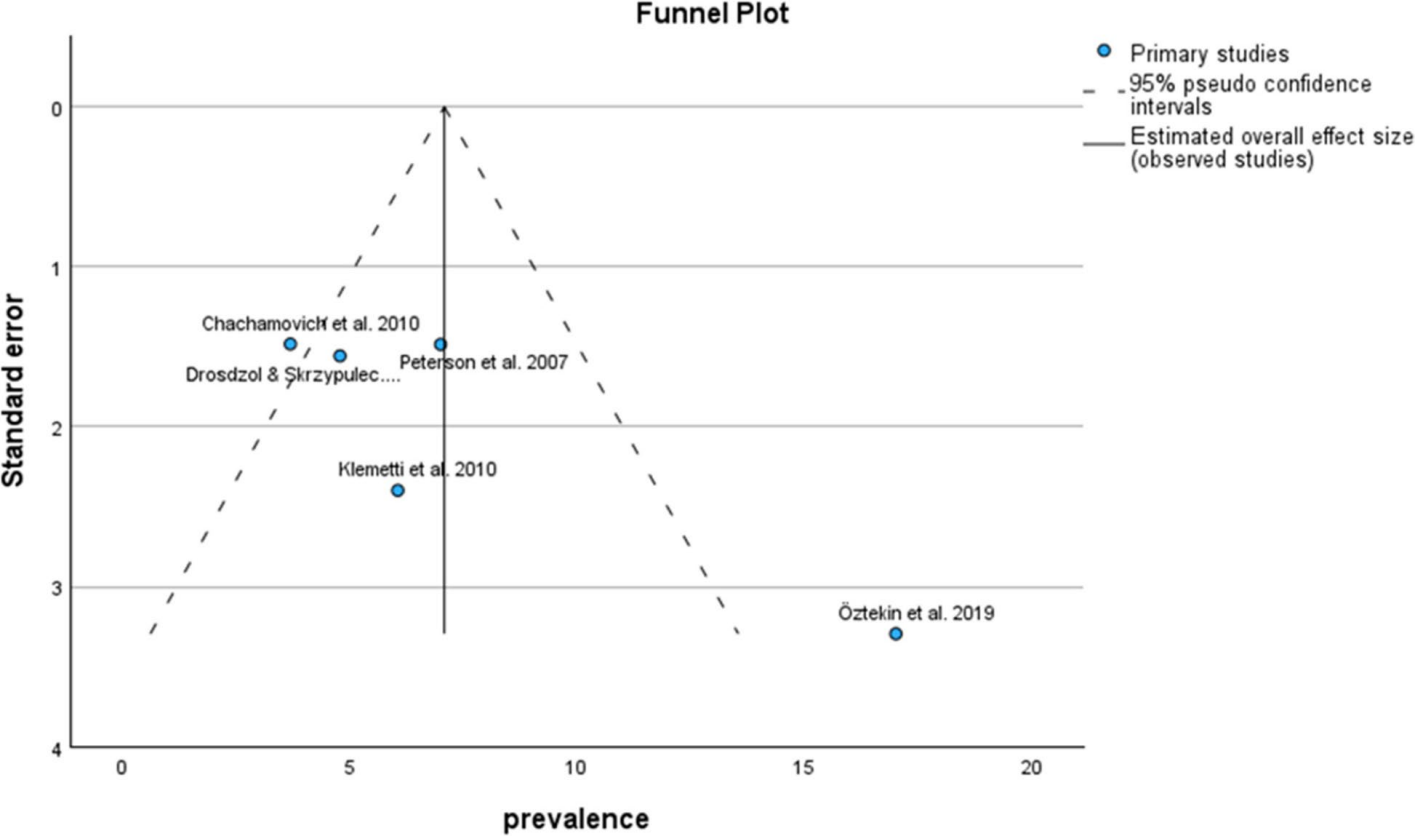
Funnel plot of pooled prevalence of anxiety symptoms in infertile men of the BAI subgroup

The results of the funnel plot are also shown in Fig. 9. The results of Egger's regression test, with a t-value of 0.832 and p-value = 0.467, suggest no significant evidence of publication bias.

### SAS

The Self-Rating Anxiety Scale (SAS) [56] is a widely used tool for assessing anxiety levels in patients with anxiety-related symptoms. The SAS test follows a self-administered format in which respondents rate each item on a 4-point scale ranging from "none of the time" to "most of the time". The scale consists of 20 questions, with 15 questions assessing increasing levels of anxiety and 5 questions evaluating decreasing levels of anxiety [57]. The Cronbach's alpha coefficient for this scale has been reported to be greater than 0.80 [57–59], and it has demonstrated good convergent and divergent validity [57].

**Fig. 10:**
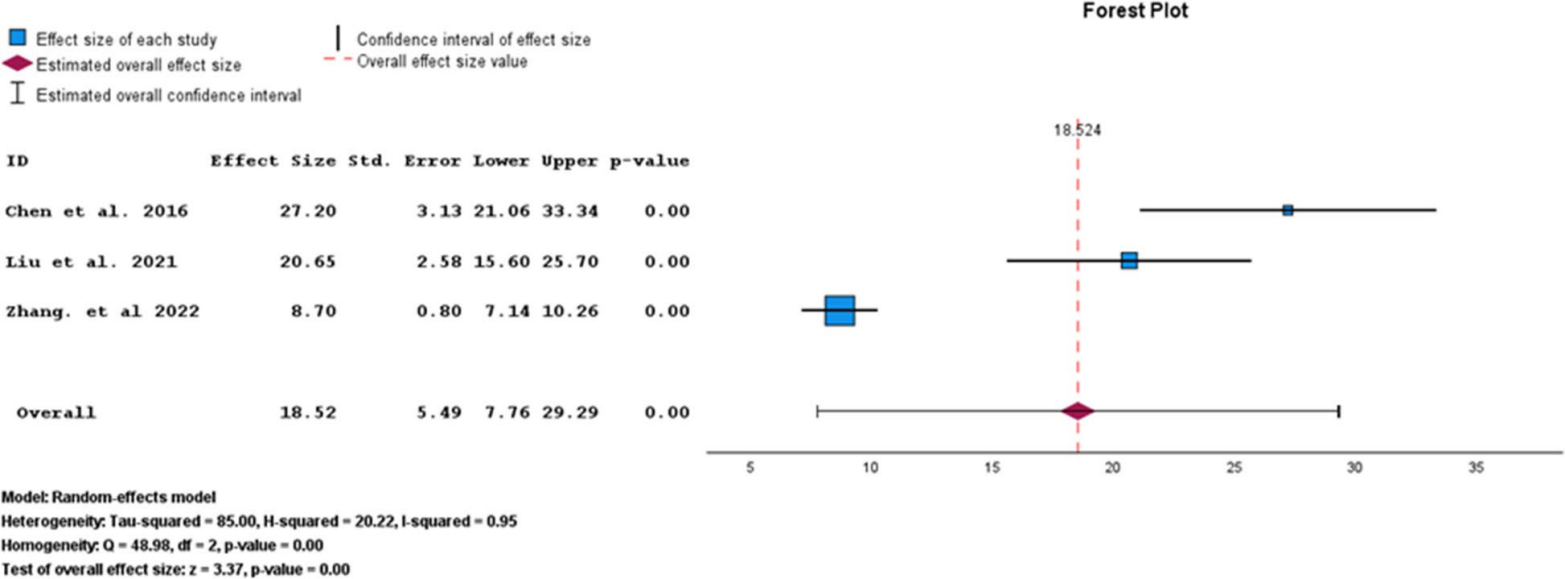
Pooled prevalence of anxiety symptoms in infertile men of the SAS subgroup

Based on the SAS subgroup results, the pooled prevalence of anxiety symptoms in infertile men was 18.52% (95% CI: 7.76–29.29) (Fig. 10).

**Fig. 11:**
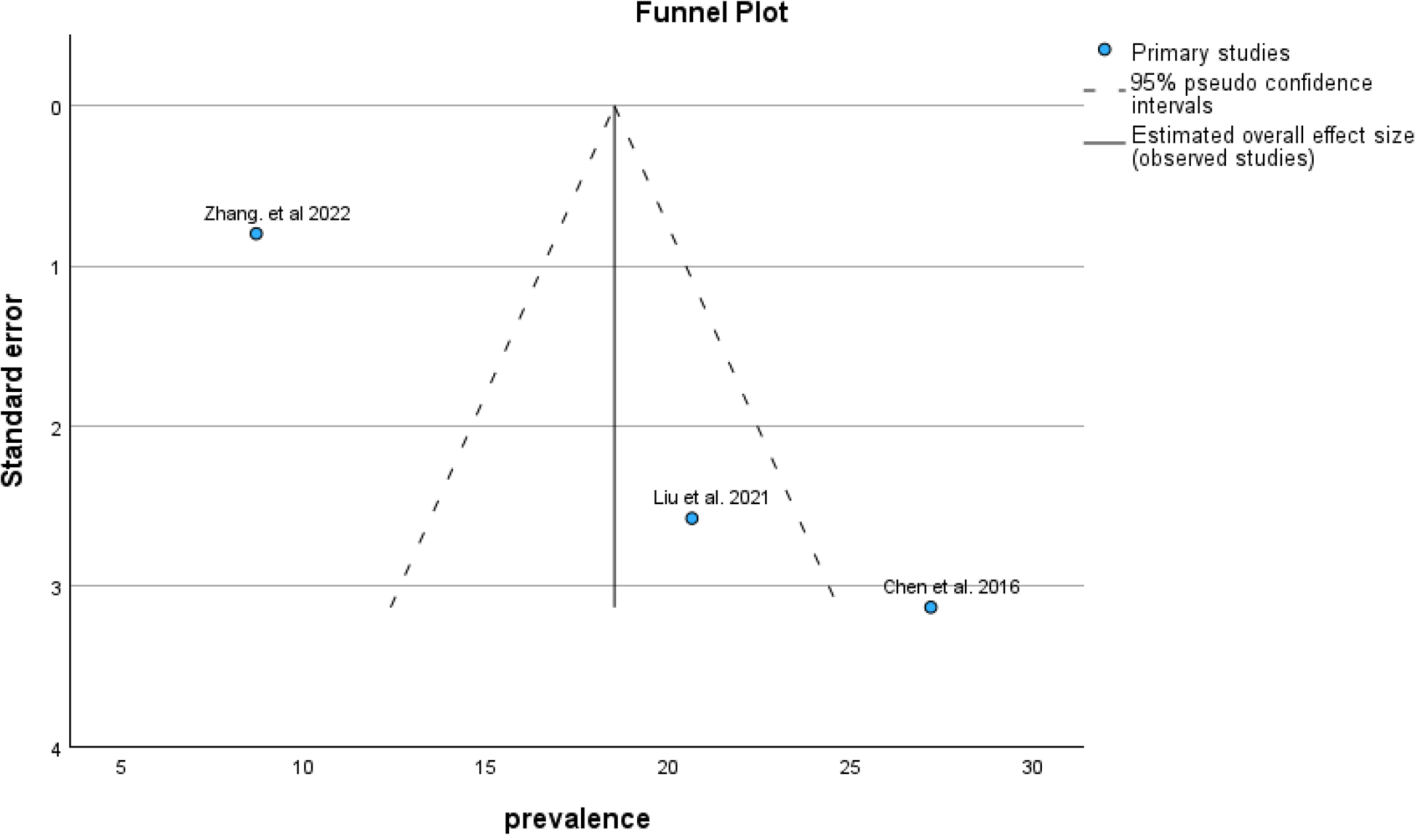
Funnel plot of pooled prevalence of anxiety symptoms in infertile men of the SAS subgroup

The results of the funnel plot are also shown in Fig. 11. The results of Egger's regression test, with a t-value of 2.045 and *p*-value = 0.290, show no significant evidence of publication bias.

### DASS

The Depression, Anxiety, and Stress Scale (DASS) was developed by Lovibond and Lovibond in 1995 [60]. The scale consists of 21 questions divided into three sections of Depression, Anxiety, and Stress, with each section containing 7 questions. The Likert scale used in the DASS ranges from 0 to 3, with the following response options: Did not apply to me at all—NEVER (0), applied to me to some extent, or some of the time—SOMETIMES (1), applied to me to a considerable extent, or a good part of the time—OFTEN (2), and applied to me very much, or most of the time—ALMOST ALWAYS (3). The scale is a shortened version of 42 questions of this tool, and after completing the scale, the scores are doubled to assess the situation of individuals over the past week [61]. The tool has been translated into several languages worldwide and has demonstrated validity and reliability in several studies [62–64].

**Fig. 12:**
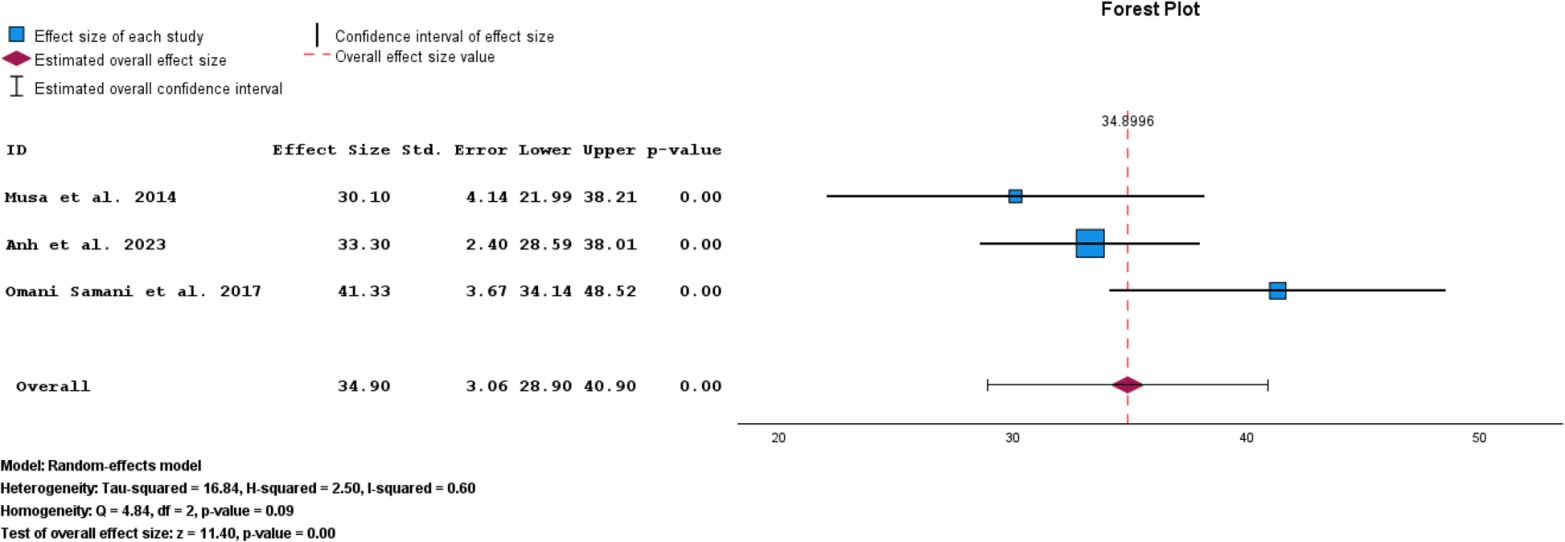
Pooled prevalence of anxiety symptoms in infertile men of the DASS subgroup

Based on the DASS subgroup results, the pooled prevalence of anxiety symptoms in infertile men was 34.90% (95% CI: 28.90–40.90) (Fig. 12).

**Fig. 13:**
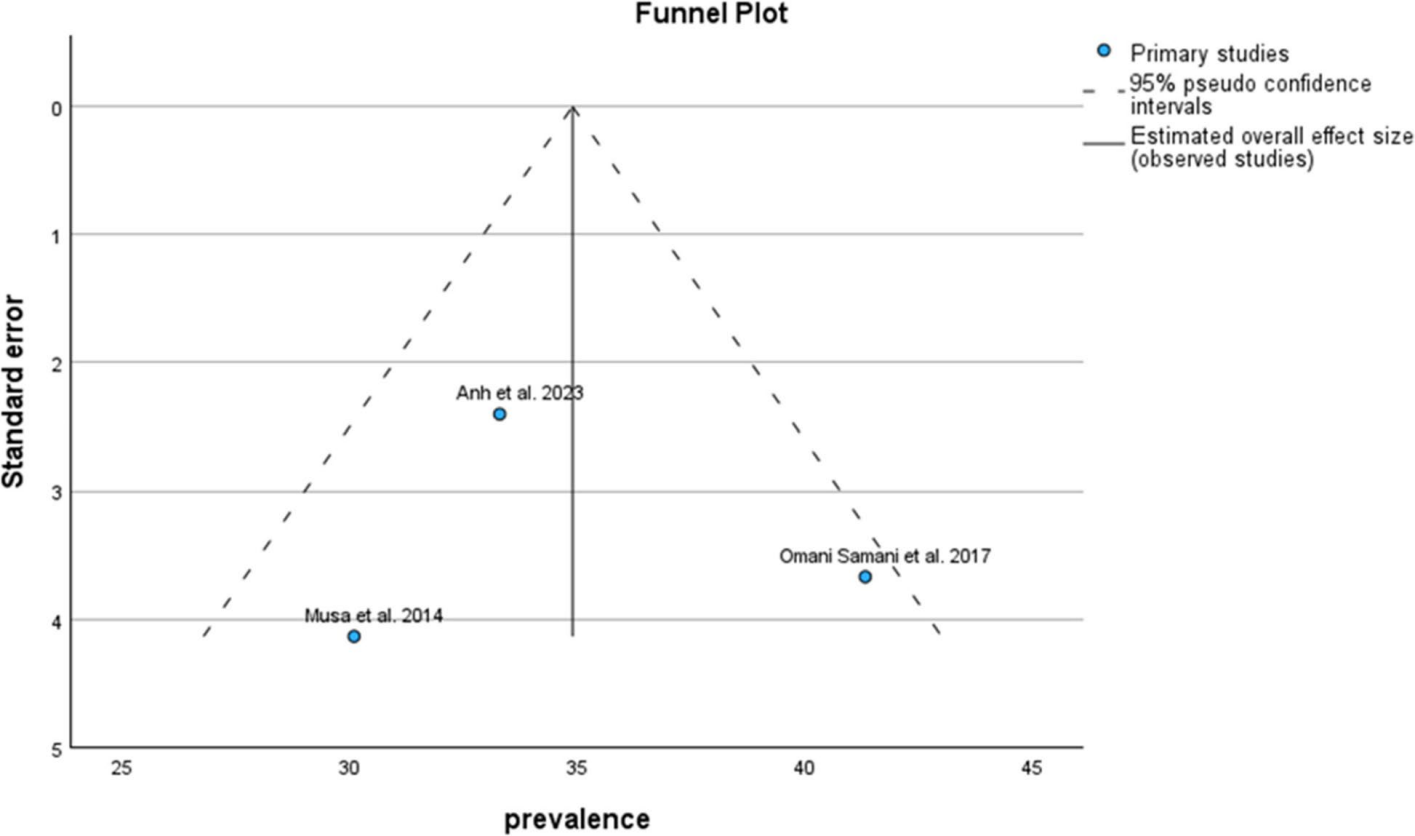
Funnel plot of pooled prevalence of anxiety symptoms in infertile men of the DASS subgroup

The results of the funnel plot are also shown in Fig. 13. The results of Egger's regression test, with a t-value of 1.618 and *p*-value = 0.352, suggest no significant evidence of publication bias.

## Discussion

This systematic review and meta-analysis investigated the global prevalence of anxiety in infertile men. This disorder is known as the most prevalent mental disorder in the world [65]. According to the results of this study, the highest and lowest prevalence of anxiety in these men were 34.9% and 7.08%, respectively, in the studies conducted using the DASS and BAI tools, which is higher than the prevalence of anxiety in the normal population and individuals without any healthy issue [66]. In a systematic review and meta-analysis study conducted in 2023, the global prevalence of anxiety was estimated to be 4.05%. According to the results of the study, the risk of anxiety was estimated to be 2.933 per 100,000 individuals in men and 4.862 per 100,000 individuals in women worldwide [67].

The desire to have children in both men and women is influenced by several factors, including individual, cultural and religious characteristics, and failure to fulfill this desire can have potential psychological consequences for individuals, such as anxiety, stress, depression, and low self-esteem [68].

According to the results of our previous meta-analysis, the estimated prevalence of anxiety in infertile women was found to be 36%, which was higher than the prevalence of anxiety in infertile men [69]. Aligned with the results of our studies, a meta-analysis study by Almutawa et al. in 2023 indicated that the prevalence of anxiety was higher in infertile women than in infertile men [70]. Although studies have indicated that infertile women often experience higher levels of anxiety than infertile men, it is important to recognize that anxiety in infertile men is a significant issue that is often overlooked during their treatment process. In some countries, coping with infertility is psychologically more difficult for men due to cultural, social and religious reasons [11].

The inability to meet personal and societal expectations regarding male fertility and reproduction is often seen as a life crisis. This is because it challenges the traditional notion of masculinity and can be associated with social stigma and significant effects on men's quality of life [71, 72]. Furthermore, many infertile men often believe that their identity and masculinity are incomplete because of the association between fertility and male sexual prowess, which causes anxiety and psychological problems in men [12].

Several factors contribute to anxiety in infertile men, including the unknown cause of infertility, the length of the treatment process, uncertainty about treatment outcomes, complex treatments, frequent doctor visits, financial stress caused by infertility, lower levels of education, and societal pressures [18, 73]. Moreover, the way men perceive themselves in the face of infertility problems and their methods of accepting infertility are important factors influencing their level of anxiety in them [74]. Infertile men, like infertile women, often experience a loss of self-confidence and inadequacy in their social and family roles. However, they try to cope with this problem and suppress their feelings through strategies such as alienation, increased participation in daily activities, a problem-solving approach, and increased support for their spouses. This can lead to underreporting of distress levels and infertility-related mental disorders in men [36, 74].

Evidence suggests that women tend to have more social support than men [75, 76]. Men's reluctance to discuss infertility with those around them, including friends, family, and counselors, may contribute to their increased stress and anxiety, and their reluctance to seek help and support to cope with these psychological distresses further exacerbates the situation. The results of a study revealed that men may not talk much about their anxiety and other psychological distress in order to provide a strong source of support for their wives during the infertility process [66]. According to studies, the most important source of emotional support for infertile couples is the spouse, and perceived social support from the spouse and other relatives is one of the influential factors for accepting infertility and coping with stressful conditions such as infertility [76–78]. Based on the results of another study, it was observed that the communication and support network of infertile men tends to diminish over time [79].

Based on the results of our previous study, the prevalence of anxiety among infertile women in low-income countries was estimated to be twice as high as that in high-income countries [69]. The results of the present study also revealed that among infertile men, the lowest prevalence of anxiety was observed among Canadian participants, with a rate of 3.7%, whereas the highest prevalence of anxiety was found among Iranian infertile men, with a rate of 42.62%. Consistent with the present study, Javaid et al. [67] indicated that Iran was the third country in the world with a high prevalence of anxiety [80]. This can be due to the fact that in many developing countries, little attention is paid to the psychological well-being of patients when it comes to dealing with infertility issues [11]. Moreover, the socio-economic situation and the availability of healthcare facilities are among the factors that influence the prevalence of anxiety in infertile individuals. Given that men are the main providers of financial resources for infertility treatment, their lack of financial resources can lead to increased psychological pressure, and this psychological burden can significantly affect their quality of life, resulting in more anxiety and psychological distress in them [81]. Thus, providing universal access to and insurance coverage for infertility services is recognized as an important policy goal for many health systems and governments.

The results of several studies have demonstrated the negative impact of mental distress on parameters that predict the success of assisted reproductive methods. In 2017, Wdowiak et al. indicated in their study that anxiety and depression in infertile individuals were associated with higher prolactin and cortisol levels, lower sex hormone-binding globulin (SHBG) and dehydroepiandrosterone sulfate (DHEA-S) secretion, and lower sperm volume and quality [82]. Therefore, given the impact of mental distress on the success of assisted reproductive methods, as well as the results of Fisher and Hammarberg's study indicating that men are more likely to seek emotional support from infertility physicians than from mental health professionals, self-help groups, or friends [83], it is crucial to integrate psychological counseling into the treatment process. The treatment team should consider the diagnosis, implementation, and evaluation of anxiety reduction interventions throughout the entire treatment process.

### The limitations and strengths

A limitation of this study was the exclusion of non-English language articles and the unavailability of full-text access to certain articles. The use of different tools in different studies to measure anxiety in infertile men was another limitation of the present study. However, the estimation of the prevalence of anxiety in infertile men based on different tools can be considered as one of the strengths of the present study, because it can be used as a guide to choose the appropriate tool to measure the anxiety of infertile men in cross-sectional and interventional studies.

## Conclusion

The results of our study estimated the prevalence of prevalence of anxiety in infertile men 21.37% (95% CI: 15.73–27.02). Considering the prevalence of anxiety in infertile men, this issue needs attention and planning to reduce the anxiety of this group of men.

### Supplementary Information

Supplementary Material 1.

Supplementary Material 2.

## Data Availability

All data related to this review is included in the result section of the manuscript. If any further data is needed it can be accessible via the corresponding author on request.

## Abbreviations

WHO: World Health Organization
NOS: The Newcastle–Ottawa Scale
PRISMA: Preferred Reporting Items for Systematic Reviews and Meta-Analyses
NA: Not reported
STAI-T: Spielberger Trait Anxiety Inventory
MINI: Mini International Neuropsychiatric Interview
HADS: Hospital Anxiety and Depression Scale
BAI: Beck anxiety inventory
DASS: Depression Anxiety Stress Scales
SAS: The Self-Rating Anxiety Scale
PRIME-MD: Primary Care Evaluation of Mental Disorders
SA-45: Symptom Assessment-45 Questionnaire
SHBG: Sex Hormone Binding Globulin
DHEA-S: Deydroepiandrosterone-Sulfate
