# Supplementary material for: Prevalence of anxiety symptoms in infertile men: a systematic review and meta-analysis
Source: BMC Public Health. 2024 Jul 6;24:1805. doi: 10.1186/s12889-024-19299-8 (PMC11227185; doi:10.1186/s12889-024-19299-8)
Supplement: Supplementary file 1 — Supplementary Material 1. [file 12889_2024_19299_MOESM1_ESM.doc]

**Appendix 1: Search strategy**

| **PubMed** |
| --- |
| (((((((((((((((((((((((Anxiety[Title/Abstract]) OR (Angst[Title/Abstract])) OR (Social Anxiety[Title/Abstract])) OR (Anxieties, Social[Title/Abstract])) OR (Anxiety, Social[Title/Abstract])) OR (Social Anxieties[Title/Abstract])) OR (Hypervigilance[Title/Abstract])) OR (Nervousness[Title/Abstract])) OR (Anxiousness[Title/Abstract])) AND (Infertility[Title/Abstract])) OR (Sterility, Reproductive[Title/Abstract])) OR (Sterility[Title/Abstract])) OR (Reproductive Sterility[Title/Abstract])) OR (Subfertility[Title/Abstract])) OR (Sub-Fertility[Title/Abstract])) AND (Prevalence[Title/Abstract])) OR (Prevalences[Title/Abstract])) OR (Period Prevalence[Title/Abstract])) OR (Period Prevalences[Title/Abstract])) OR (Prevalence, Period[Title/Abstract])) OR (Point Prevalence[Title/Abstract])) OR (Point Prevalences[Title/Abstract])) OR (Prevalence, Point[Title/Abstract])) OR (Epidemiology[Title/Abstract])  **N=2391** |
| **Scopus** |
| ( TITLE-ABS-KEY ( prevalence ) OR TITLE-ABS-KEY ( epidemiology ) AND TITLE-ABS-KEY ( anxiety ) OR TITLE-ABS-KEY ( hypervigilance ) OR TITLE-ABS-KEY ( nervousness ) OR TITLE-ABS-KEY ( social AND anxiety ) AND TITLE-ABS-KEY ( infertility ) OR TITLE-ABS-KEY ( sterility ) OR TITLE-ABS-KEY ( reproductive AND sterility ) OR TITLE-ABS-KEY ( subfertility ) OR TITLE-ABS-KEY ( sub-fertility ) )  **N= 449** |
| **ISI** |
| ((TS=(infertility)) AND TS=(anxiety)) AND TS=(prevalence)  **N=295** |
