# Supplementary material for: Prevalence of anxiety symptoms in infertile men: a systematic review and meta-analysis
Source: BMC Public Health. 2024 Jul 6;24:1805. doi: 10.1186/s12889-024-19299-8 (PMC11227185; doi:10.1186/s12889-024-19299-8)
Supplement: Supplementary file 2 — Supplementary Material 2. [file 12889_2024_19299_MOESM2_ESM.doc]

**Appendix 2: Quality Assessment**

Modified Newcastle-Ottawa scoring guide.

**(1) Representativeness of the sample:**

1 point: Population contained a mixture of specialties at multiple sites.

0 points: Population contained a single specialty at a single site.

**(2) Sample size:**

1 point: Sample size was 200 and greater than 200 participants.

0 points: Sample size was less than 200 participants or a convenience sample.

**(3) Non-respondents:**

1 point: Comparability between respondent and non-respondent characteristics was established, and the response rate was satisfactory.

0 points: The response rate was unsatisfactory, the comparability between respondents and non-respondents was unsatisfactory, or there was no description of the response rate or the characteristics of the responders and the non-responders.

**(4) Ascertainment of anxiety:**

1 point: Validated measurement tool using a validated cutoff score or clinical interview.

0 points: Non-validated measurement tool, or validated measurement tool with non-valid cutoff score, or 2-item PRIME-MD (scored as such due to its low specificity).

**(5) Quality of descriptive statistics reporting:**

1 point: Reported descriptive statistics to describe the population (*e.g.*, age, sex) with proper measures of anxiety (*e.g.*, standard deviation, standard error, range, precentage).

0 points: Descriptive statistics were not reported, were incomplete, or did not include proper measures of anxiety.

**Legend:** This scale, the scoring of which ranges from 0 to 5, assesses quality in several domains: sample representativeness and size, comparability between respondents and non-respondents, ascertainment of depressive symptoms, and statistical quality. Studies were judged to be of low risk of bias (≥3 points) or high risk of bias (<3 points).

**Total = /5**

|  | **Results of Newcastle-Ottawa Risk of Bias Assessment** | | | | | | |
| --- | --- | --- | --- | --- | --- | --- | --- |
|  | **Study ID** | Representativeness | Size | Comparability | Outcome | Statistics | Total |
| **1** |  | 0 | 1 | 1 | 1 | 1 | 4 |
| **2** |  | 0 | 0 | 1 | 1 | 1 | 3 |
| **3** |  | 1 | 0 | 0 | 1 | 1 | 3 |
| 4 |  | 1 | 1 | 0 | 1 | 1 | 4 |
| **5** |  | 0 | 0 | 1 | 1 | 1 | 3 |
| **6** |  | 0 | 0 | 1 | 1 | 1 | 4 |
| **7** |  | 1 | 1 | 1 | 1 | 1 | 5 |
| 8 |  | 1 | 1 | 0 | 0 | 1 | 3 |
| **9** |  | 0 | 1 | 1 | 1 | 1 | 4 |
| **10** |  | 0 | 1 | 1 | 1 | 1 | 4 |
| **11** |  | 1 | 1 | 1 | 1 | 1 | 5 |
| **12** |  | 0 | 1 | 1 | 1 | 1 | 4 |
| **13** |  | 0 | 0 | 1 | 1 | 1 | 3 |
| **14** |  | 0 | 1 | 1 | 1 | 1 | 4 |
| **15** |  | 0 | 1 | 0 | 1 | 1 | 3 |
| **16** |  | 0 | 0 | 1 | 1 | 1 | 3 |
| **17** |  | 0 | 1 | 0 | 1 | 1 | 3 |
| **18** |  | 0 | 1 | 1 | 1 | 1 | 4 |
| **19** |  | 0 | 0 | 1 | 1 | 1 | 3 |
| **20** |  | 0 | 0 | 1 | 1 | 1 | 3 |
| **21** |  | 0 | 1 | 1 | 1 | 1 | 4 |
| **22** |  | 0 | 0 | 1 | 1 | 1 | 3 |
| **23** | **(Drosdzol and Skrzypulec, 2009)** | 1 | 1 | 1 | 1 | 1 | 5 |
| **24** |  | 1 | 1 | 1 | 1 | 1 | 5 |
| **25** |  | 1 | 1 | 1 | 0 | 1 | 4 |
| **26** |  | 0 | 1 | 1 | 1 | 1 | 4 |
| **27** | **(Haimovici et al., 2018)** | 0 | 0 | 1 | 1 | 1 | 3 |
